# Clustering Runners’ Response to Different Midsole Stack Heights: A Field Study

**DOI:** 10.3390/s24144694

**Published:** 2024-07-19

**Authors:** Jannik Koegel, Stacy Huerta, Markus Gambietz, Martin Ullrich, Christian Heyde, Eva Dorschky, Bjoern Eskofier

**Affiliations:** 1 Machine Learning and Data Analytics Lab, Department Artificial Intelligence in Biomedical Engineering, Friedrich-Alexander-Universität Erlangen-Nürnberg (FAU), 91052 Erlangen, Germany; stacy.huerta@fau.de (S.H.); eva.dorschky@fau.de (E.D.); 2 adidas AG, 91074 Herzogenaurach, Germany; martin.ullrich@adidas.com (M.U.); christian.heyde@adidas.com (C.H.); 3 Autonomous Systems and Mechatronics, Friedrich-Alexander-Universität Erlangen-Nürnberg (FAU), 91052 Erlangen, Germany; markus.gambietz@fau.de

**Keywords:** running shoes, running biomechanics, wearable sensor, cluster analysis, principal component analysis

## Abstract

Advanced footwear technology featuring stack heights higher than 30 mm has been proven to improve running economy in elite and recreational runners. While it is understood that the physiological benefit is highly individual, the individual biomechanical response to different stack heights remains unclear. Thirty-one runners performed running trials with three different shoe conditions of 25 mm, 35 mm, and 45 mm stack height on an outdoor running course wearing a STRYD sensor. The STRYD running variables for each participant were normalized to the 25 mm shoe condition and used to cluster participants into three distinct groups. Each cluster showed unique running patterns, with leg spring stiffness and vertical oscillation contributing most to the variance. No significant differences were found between clusters in terms of body height, body weight, leg length, and running speed. This study indicates that runners change running patterns individually when running with footwear featuring different stack heights. Clustering these patterns can help understand subgroups of runners and potentially support running shoe recommendations.

## 1. Introduction

The introduction of advanced footwear technology (AFT) in 2017 started with the Nike Vaporfly 4%, which has been proven to improve running economy in elite [1,2,3] and recreational runners [4]. While there is no known comprehensive biomechanical explanation for the improvements caused by shoes featuring AFT [5], several studies have been looking into the different AFT characteristics independently.

The three major characteristics of AFT are a high stack height (>30 mm), a curved rocker geometry of the midsole, and a stiff element embedded into the midsole [6]. The stack height of shoes raised particular interest, as it was quickly regulated for official races by the World Athletics in 2021 to not exceed 40 mm [7].

Increasing the shoe midsole thickness inherently increases the overall weight. Increased footwear weight leads to higher oxygen consumption and therefore lowers running economy [8,9]. Other effects that can be created with higher midsoles could potentially counteract the detrimental effect of a heavier shoe. An example could be the ‘teeter-totter-effect’, where during the push-off phase the generated force in the forefoot creates a reaction force at the heel in the upwards direction. This is possible through the midsole curvature in the forefoot, acting as a fulcrum and therefore contributing to better running economy [10]. Barrons et al. 2023 [11] could not find an influence of stack height on running economy using weight-matched shoes with thermoplastic polyester elastomer (TPEE) midsoles ranging from 35 to 50 mm in 5 mm increments. This was confirmed by Bertschy et al., 2023 [12], who used shoes with two different foams with the lowest featuring a stack height of 30 mm and the highest featuring a stack height of 60 mm. Although no significant difference was found in running economy, it was stated that the more compliant and resilient foam could decrease metabolic power.

As a biomechanical response, it has been shown that a higher stack height leads to reduced loading rates, a longer ground contact time, a longer stride length, and a lower cadence [13,14]. The transfer of these findings to AFT shoes is limited though, as stack height was either not systematically controlled or the highest stack height was lower than 30 mm.

Recent studies have focused on understanding the unique responses of runners under varying shoe conditions. The use of AFT compared to more conventional or racing flat running shoes has been shown to improve running economy only for a limited number of individuals. Furthermore, the magnitude of the benefit appears to be highly individual as well. This was found to be true for runners from elite and recreational levels [15], while metabolic savings of 4% or more can only be expected for around 25% of runners [16].

All of the studies have been conducted in a laboratory environment mainly performed on treadmills, and, to the best of our knowledge, research has never focused on the individual responses of runners other than metabolic costs when running in shoes featuring different stack heights. The purpose of this study was to investigate individual biomechanical running responses with the aim of finding distinctive clusters using a wearable sensor in the field. It was hypothesized that runners would change running patterns differently when running in shoes with varied midsole stack heights. Clustering these running patterns would facilitate the distinction and comprehension of subgroups of runners.

## 2. Materials and Methods

### 2.1. Shoes

**Figure 1:**
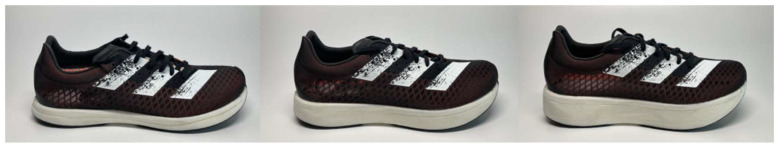
Shoe conditions displayed by stack height in ascending order, from left to right (25 mm, 35 mm, 45 mm).

Three different experimental shoe conditions were specifically developed and manufactured for this study. All shoes featured the same upper, outsole, and carbon plate embedded into the midsole. While in each shoe condition the midsole foam (TPEE) was identical, the stack heights in the three shoe conditions were 25 mm, 35 mm, and 45 mm (see Figure 1). Shoes were produced in sizes UK 8.5 and UK 10.5 to increase the potential participant pool.

### 2.2. Footwear and Mechanical Testing Shoes

The shoes were evaluated for rearfoot and forefoot cushioning, energy return, and forefoot bending stiffness (see Table 1) using a servo-hydraulic testing machine (Instron^®^, Norwood, MA, USA). Cushioning and energy return tests followed the same test methodology described by Dorschky et al. 2019 [17], with a maximal load of 2000 N for the UK 8.5 shoes and 2250 N for the UK 10.5 shoes for both forefoot and rearfoot testing. The stiffness was calculated at 30–50% of the maximal load. The bending stiffness was captured through a three-point bending test, with a maximum bending angle of 40 degrees.

### 2.3. Human Participants

Thirty-one male subjects (31.13 ± 6.92 y, 181.34 ± 5.71 cm, 72.46 ± 5.95 kg, 51.58 ± 18.97 km/week) participated in this study. Inclusion criteria for participants were having a shoe size that matched the footwear used in the study, a documented history of running 10 km in under 44 min (35.46 ± 2.89 min, *n* = 24) and/or 5 km in under 22 min (16.66 ± 1.67 min, *n* = 25), and no self-reported major musculoskeletal injuries of the lower extremities in the past 6 months. Prior to testing, body height and leg lengths were measured, and body weight was captured (seca 634, Seca GmbH & Co. KG, Hamburg, Germany). All participants provided informed consent, and this study was approved by the ethics committee of the Medical Faculty at the FAU Erlangen-Nürnberg, Germany (Ref.-No.: 22-437-B).

### 2.4. Study Protocol

Subjects were instructed to complete an 8-min warm up in their own shoes following their individual usual warm-up routine. After the 8-min warm-up period, each participant also completed two 60 m strides for a final muscle activation. Upon completion of the warm up, each participant was equipped with a GARMIN Forerunner 245 Music (Garmin Inc., Olathe, KS, USA) for starting and stopping data recording and a STRYD sensor (Stryd Inc., Boulder, CO, USA) model 26 with firmware version 2.1.30, which was attached to the right shoe covering the three most distal lace crossings. The STRYD sensor is a commercially available wearable device featuring an inertial measurement unit to inform runners about specific running parameters of their runs. Testing took place in two different locations, both featuring a flat running course with a concrete surface. Subjects ran a 1.2 km distance in each experimental shoe condition featuring a randomized order. For each run, subjects were instructed to set the individual effort to competing in a 10 km race, and subjects were ordered to not check running speed during and after the trials. In between runs, subjects had a 5-min break to recover and to switch shoes.

### 2.5. Data Analysis

The data output from the sensor consisted of eight different variables: cadence, speed, flight time, ground contact time, stride length, leg spring stiffness, vertical oscillation, and peak ground reaction force, with a temporal resolution of 1 Hz. To consider the individual body weight and leg length of each participant, peak ground reaction force, leg spring stiffness, vertical oscillation, flight time, and stride length were recalculated using the methodology from Morin et al. 2005 [18]. For all further calculations, the first 500 m and last 100 m of each run were cut off to eliminate the shoe adaptation phase and any speed inconsistencies while approaching the finish line, leaving 600 m of running data for further analysis.

For each participant, the data collected from the sensor were averaged for the remaining 600 m and underwent normalization, calculating the percentage difference of the 35 mm shoe and the 45 mm shoe with respect to the 25 mm shoe. With the normalized dataset, a principal component analysis (PCA) was performed with the goal of reducing the number of dimensions. The number of dimensions for further analysis was determined based on the criterion that more than 80% of the data variance could be explained by the retained principal components. The principal component values were used as input for clustering with Ward’s method [19], aiming to generate three clusters, which were chosen after using the Silhouette and Elbow methods to determine the optimal number of clusters. It was evaluated if the average running trial speed or the anthropometric data, which were recorded from the participants, had a significant influence on the cluster classification using a one-sided ANOVA. Normal distribution of the data was confirmed with a Shapiro–Wilk test (α > 0.05), and the level of significance for the ANOVA was set to α = 0.05. Finally, average values and standard deviations of the sensor outputs were calculated for each cluster to describe their respective running response to the shoe conditions. Only the parameter contributing most to the variance of the dataset and explaining more than 80% of variance of both principal components was considered in the display of the results. The complete overview of parameters can be found in Appendix A.

## 3. Results

**Figure 2:**
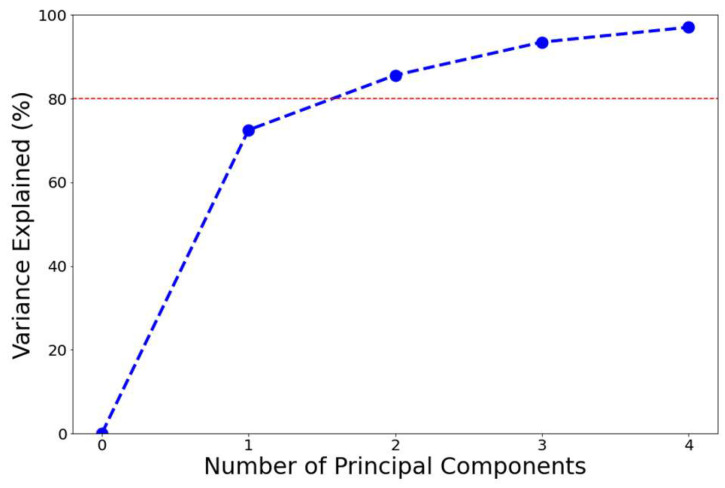
Cumulative proportion of variance explained by the number of dimensions. The red line indicates the 80% variance explained threshold.

Figure 2 shows the cumulative proportion of variance explained by the respective number of dimensions. With two dimensions, the explanation of variation reached 85.6% and therefore passed the set threshold of 80%. The following clustering was therefore conducted using the principal component values of the two retained dimensions.

**Figure 3:**
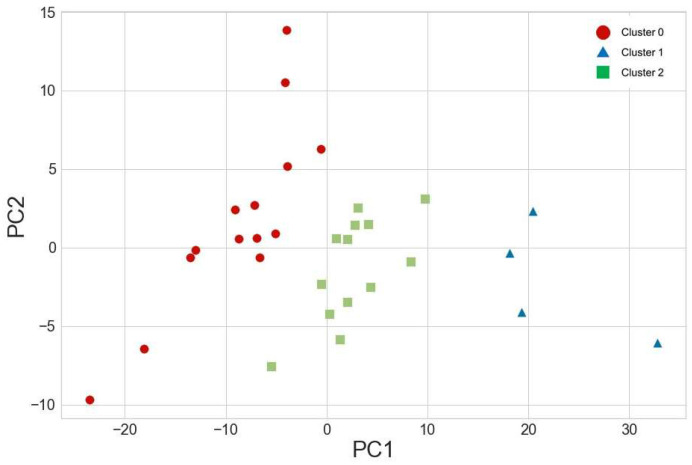
Cluster analysis results based on two principal components (PC1 and PC2) using Ward’s method [19].

The result of the clustering analysis with Ward’s method [19] is displayed in Figure 3. The distribution of participants between clusters differs with cluster 0 containing 14 subjects, cluster 1 including 4 subjects, and cluster 2 having 13 subjects. No significant differences were observed between clusters in terms of body height, body weight, leg length, or average trial running speed among the participants (see Table 2).

**Figure 4:**
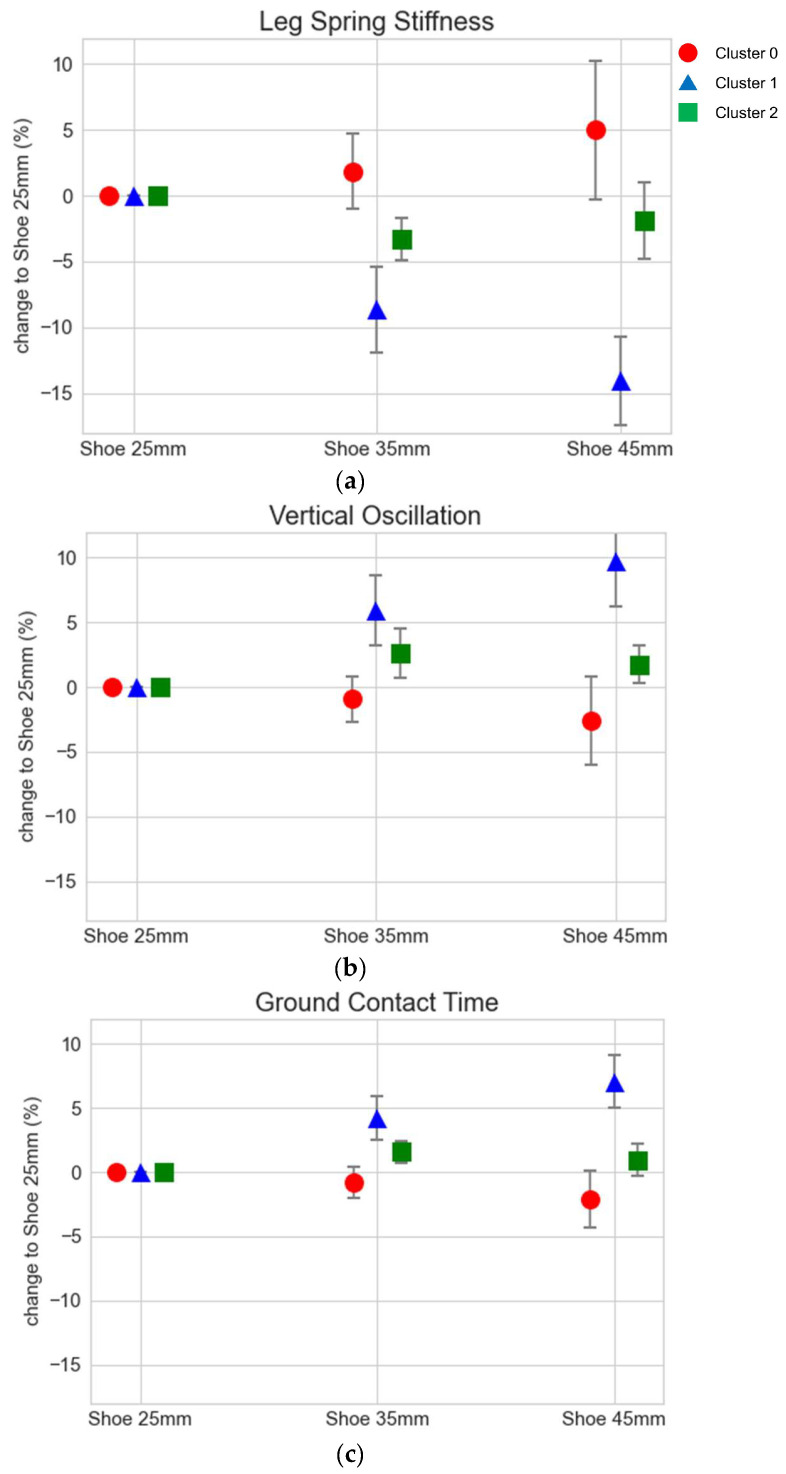

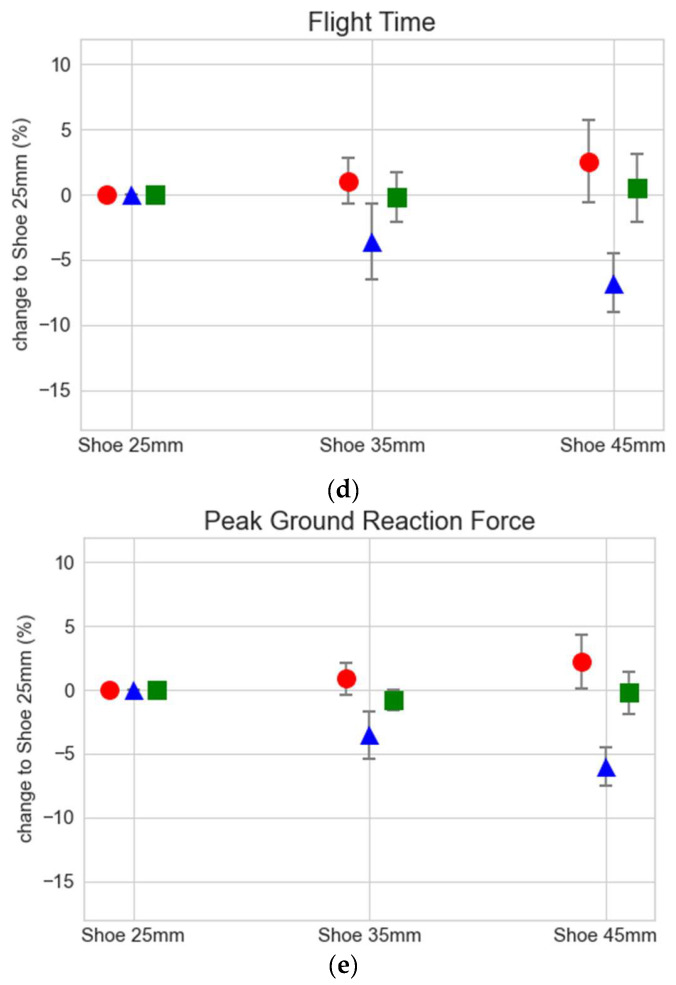
Mean and standard deviation of running parameters normalized to the 25 mm shoe across clusters: (**a**) leg spring stiffness, (**b**) vertical oscillation, (**c**) ground contact time, (**d**) flight time, and (**e**) peak ground reaction force.

The average values and standard deviations of the recorded running parameters of each cluster are displayed in Figure 4 in descending order of variance contribution. The numeric values are listed in Table A1 in Appendix A. Cluster 0 showed a gradual increase in leg spring stiffness and a decrease in both vertical oscillation and ground contact time when running in shoes with increasing stack heights. The flight times were longer and the peak ground reaction force was higher for the participants in this cluster. Cluster 1 gradually decreased in leg spring stiffness but increasd in vertical oscillation and ground contact time using the shoes with 35 mm and 45 mm stack heights. In this group, the flight times and peak ground reaction forces were both reduced with higher stack heights. The leg spring stiffness of Cluster 2 decreased using the 35 mm shoe but increased 1.4% in the 45 mm shoe compared to the 35 mm shoe. The vertical oscillation and ground contact time both increased for cluster 2 and had their peak with the 35 mm shoe. Furthermore, the flight time was reduced with the 35 mm shoe but showed an increase with the 45 mm shoe. The peak ground reaction forces were reduced in both shoe conditions with a higher stack height by less than 1%.

## 4. Discussion

The results of this study show that runners respond differently when running in shoes featuring varied stack heights. Clustering methods based on sensor outputs were used to create distinct groups showing different response patterns, which confirmed the hypothesis of this study. While subjects in cluster 2 showed only small changes in the recorded running parameter, cluster 0 and cluster 1 had a contrary pattern. The difference in responses aligns with the findings that runners show individual benefits using AFT [15,16].

Interpreting the response patterns of the three clusters in this study, cluster 2 could be seen as “non-responders”, as runners in this cluster showed minimal changes in the recorded parameters when running in the different shoes. Running with less vertical oscillation and higher leg spring stiffness is associated with better running economy [20]. This could potentially define runners in cluster 0 as positive (more economical) and runners in cluster 1 as negative (less economical) responders to increased stack heights. The considerably bigger size of the cluster with potential positive responders could explain the effect that running with AFT shoes on average significantly benefits running economy [1,2,3,4]. While this would need to be verified with oxygen consumption measurements, these findings could potentially help to recommend to runners the most economical shoe condition using running parameters from wearable sensors.

In this study, no significant influence was found on the classification of runners through running speed or simple anthropometrical data like body height, body weight, and leg length. Further research should investigate the reasons why runners react differently to shoes featuring different stack heights. Increasing participant numbers could enable further machine learning methods trying to predict cluster classification.

The shoes that have been used in this study have not been weight-matched because it represents the realistic weight increase when using higher stack heights, as no oxygen consumption measurement has been performed. Additionally, the resulting maximum weight difference of 44 g should have had limited effects on the running performance and response [8,9]. The increase in stack height also directly influences the cushioning and energy return properties of running shoes. Using the same foam at higher stack heights consequently decreases cushioning stiffness and increases absolute energy return. Therefore, the running responses cannot be solely isolated for the stack height, which is a limitation in interpreting these results. In the future, foams could be specifically engineered for the different shoe versions to better isolate the differences to one specific shoe parameter. The bending stiffness in between the shoes was very similar, despite their having substantially different stack heights. This shows that the carbon plate as the stiffening element is the dominant feature contributing to the bending stiffness and secures bending stiffness comparability in between shoes when using different foam geometries.

The runners in this study were instructed to run at a self-selected speed that reflected their subjective effort in a 10 km race, without the possibility of checking their pace. This could have led to speed differences between the individual runs with effects on speed-dependent variables like stride length. Having speed-controlled runs, on the other hand, would have taken away the option to vary the running speed of the runner based on their subjective effort. In this study, it was decided to take an effort-based approach, allowing potential speed changes using the different shoes and taking it into consideration as a parameter in the analysis. As speed differences between the shoe conditions and therefore contributions to the data variance were minimal, ranging on average between −2.3% and +1.9% (Appendix A, Table A1), it was not considered in the display of results.

To the best of our knowledge, this is the first study that investigated shoe properties and clustering running pattern responses in the field with a commercially available sensor. The reliability of the STRYD sensor has been proven accurate for ground contact time, leg spring stiffness, flight time, stride length, and cadence [21,22], but it is yet to be validated for vertical oscillation and peak ground reaction force. Having the possibility to use the individual’s leg length could give a more individualized approach in the results for the end user and would be recommended as an option in further software development of wearable sensors.

Using a wearable sensor to collect measurements in the field provides researchers with the possibility of creating large data sets for limited costs in a natural environment. Still, limitations like reduced parameter availability and accuracy need to be considered when designing studies “in the wild” to generate meaningful insights [23].

This study could provide the blueprint for runners to evaluate their running response to different footwear models themselves. Running with different footwear models using the same running course and subjective effort, the comparison of vertical oscillation and leg spring stiffness between runs could potentially support the choice of footwear providing better running economy.

## Figures and Tables

**Table 1:** Mechanical test results of all shoe conditions.

Size (UK)	Stack Height (mm)	Weight (g)	Cushioning (N/mm) Rearfoot/Forefoot	Energy Return (Rel) (%)	Energy Return (Abs) (J)	Bending Stiffness (Nm/°)
8.5	25.0	186.0	128/136	83.0	8.0	0.22
8.5	35.0	211.0	86/100	82.0	12.4	0.20
8.5	45.0	229.0	66/77	83.0	16.4	0.20
10.5	25.0	202.2	144/155	80.0	9.6	0.22
10.5	35.0	229.0	99/108	82.0	14.5	0.23
10.5	45.0	246.0	76/83	83.0	18.9	0.21

**Table 2:** Mean ± standard deviation of anthropometrical characteristics and running speed across clusters.

	Cluster 0	Cluster 1	Cluster 2	F-Score	*p*-Value
Body height (cm)	180.3 (±5.61)	182.5 (±5.07)	182.0 (±6.24)	0.40	0.674
Body weight (kg)	73.7 (±5.08)	74.3 (±4.79)	70.8 (±6.99)	0.96	0.396
Leg length (cm)	94.78 (±3.32)	95.5 (±3.42)	95.7 (±4.34)	0.19	0.832
Running speed (m/s)	4.7 (±0.57)	4.8 (±0.11)	4.9 (±0.30)	0.80	0.458

## Data Availability

Data are available upon request.

## Appendix A

**Table A1:** Mean and standard deviation (std) of all running parameters in % difference to the 25 mm shoe, separated by clusters.

		Cluster 0		Cluster 1		Cluster 2	
		Mean	std	Mean	std	Mean	std
Leg Spring Stiffness	Shoe 35 mm	1.8	2.85	−8.7	3.26	−3.3	1.63
	Shoe 45 mm	5.0	5.26	−14.1	3.36	−1.9	2.91
Vertical Oscillation	Shoe 35 mm	−0.9	1.72	5.9	2.74	2.6	1.90
	Shoe 45 mm	−2.6	3.41	9.7	3.50	1.7	1.46
Ground Contact Time	Shoe 35 mm	−0.8	1.19	4.2	1.69	1.6	0.86
	Shoe 45 mm	−2.0	2.21	7.0	2.06	−0.2	1.23
Flight Time	Shoe 35 mm	1.0	1.77	−3.6	2.91	−0.2	1.93
	Shoe 45 mm	2.5	3.17	−6.8	2.24	0.5	2.59
Peak Ground Reaction Forces	Shoe 35 mm	0.9	1.27	−3.5	1.85	−0.8	0.76
	Shoe 45 mm	2.2	2.11	−6.0	1.50	−0.2	1.69
Speed	Shoe 35 mm	0.6	1.23	−1.5	4.00	−1.0	1.88
	Shoe 45 mm	1.9	3.48	−2.3	4.15	−0.1	1.60
Stride Length	Shoe 35 mm	0.7	1.18	−1.0	2.88	−0.3	1.32
	Shoe 45 mm	2.0	2.83	−1.7	3.49	0.6	1.63
Cadence	Shoe 35 mm	−0.1	0.72	−0.5	1.48	−0.8	1.18
	Shoe 45 mm	−0.1	1.43	−0.6	1.26	−0.7	0.74
